# Prevalence of antithyroperoxidase antibodies in a multiethnic Brazilian population: The ELSA-Brasil Study

**DOI:** 10.20945/2359-3997000000122

**Published:** 2019-04-15

**Authors:** Carolina Castro Porto Silva Janovsky, Marcio Sommer Bittencourt, Alessandra C. Goulart, Itamar S. Santos, Bianca Almeida-Pititto, Paulo A. Lotufo, Isabela M. Benseñor

**Affiliations:** 1 Universidade de São Paulo Centro de Pesquisa Clínica e Epidemiológica Hospital Universitário Universidade de São Paulo São Paulo SP Brasil Centro de Pesquisa Clínica e Epidemiológica, Hospital Universitário, Universidade de São Paulo, São Paulo, SP, Brasil; 2 Universidade de São Paulo Instituto do Coração, Hospital das Clínicas Faculdade de Medicina Universidade de São Paulo São Paulo SP Brasil Instituto do Coração, Hospital das Clínicas, Faculdade de Medicina, Universidade de São Paulo (InCor- -HCFMUSP), São Paulo, SP, Brasil; 3 Universidade de São Paulo Departamento de Medicina Interna Faculdade de Medicina Universidade de São Paulo São Paulo SP Brasil Departamento de Medicina Interna, Faculdade de Medicina, Universidade de São Paulo (FMUSP), São Paulo, SP, Brasil; 4 Universidade Federal de São Paulo Departamento de Medicina Preventiva Escola Paulista de Medicina Universidade Federal de São Paulo São Paulo SP Brasil Departamento de Medicina Preventiva, Escola Paulista de Medicina, Universidade Federal de São Paulo (EPM-Unifesp), São Paulo, SP, Brasil

**Keywords:** Tthyroiditis, autoimmune, Graves disease, hypothyroidism, primary, deficiency, TSH, autoimmune diseases

## Abstract

**Objective:**

In this study, we aimed to describe the prevalence and distribution of positive antithyroperoxidase antibodies (TPOAb) according to sex, age strata, and presence of thyroid dysfunction using baseline data from the Brazilian Longitudinal Study of Adult Health (ELSA-Brasil).

**Materials and methods:**

Thyroid hormone tests were obtained from each study participant at baseline. Levels of thyroid-stimulating hormone (TSH) and free thyroxine (FT4) were measured using a third-generation immunoenzymatic assay. Antithyroperoxidase antibodies were measured by electrochemiluminescence and were considered to be positive when ≥ 34 IU/mL.

**Results:**

The prevalence of TPOAb among 13,503 study participants was 12%. Of participants with positive TPOAb, 69% were women. Almost 60% of the individuals with positive TPOAb were white. The presence of positive TPOAb was associated with the entire spectrum of thyroid diseases among women, but only with overt hyperthyroidism and overt hypothyroidism in men.

**Conclusion:**

The distribution of positive TPOAb across sex, race, age, and thyroid function in the ELSA-Brasil study is aligned with the worldwide prevalence of positive TPOAb reported in iodine-sufficient areas. In women, the presence of TPOAb was related to the entire spectrum of thyroid dysfunction, while in men, it was only related to the occurrence of overt thyroid disease.

## INTRODUCTION

Thyroid dysfunction is emerging as one of the most common diseases in developed countries (1-5). Although the prevalence of overt disease (hypothyroidism and hyperthyroidism) is lower than 2%, subclinical disease is far more prevalent (5-10). Subclinical hypothyroidism may be present in 4%-15% of the population (11) and is seen mainly in older women (5,6,12).

Autoimmune thyroid disease has emerged as the main cause of thyroid dysfunction after the frequency of goiter caused by iodine deficiency decreased following compulsory salt iodination in developing countries (13-16). Worldwide, the most common conditions associated with thyroid dysfunction are overt and subclinical hypothyroidism (10,17-19) due to autoimmune Hashimoto’s thyroiditis (20-22). Among other features, the presence of serum antithyroperoxidase antibodies (TPOAb) is the main diagnostic element of this condition (23-25).

The prevalence of positive TPOAb across race, gender, and age is still not well established in the general population in Brazil. Therefore, we aimed in this study to describe the prevalence and distribution of TPOAb according to sex, age strata, and presence of thyroid dysfunction using baseline data from the Brazilian Longitudinal Study of Adult Health (ELSA-Brasil).

## MATERIALS AND METHODS

This was a cross-sectional analysis using baseline data from the ELSA-Brasil, a prospective cohort study. The study design and cohort profile have been published elsewhere (26). Briefly, the study cohort comprised 15,105 active and retired civil servant employees from six different Brazilian cities (Salvador, Vitória, Belo Horizonte, Rio de Janeiro, São Paulo, and Porto Alegre) aged between 35 to 74 years at baseline. Information on sociodemographic data, personal and family history of diseases, lifestyle factors, mental health, cognitive status, and occupational exposure were assessed from August 2008 to December 2010 (27). The aim of the study was to determine the incidence of cardiovascular disease and diabetes along with factors associated with these conditions. Nonclassical risk factors possibly associated with cardiovascular diseases, such as thyroid function, were also measured.

The institutional ethics committee approved the protocol of the study, and written consent was obtained from all individuals.

### Thyroid function

Thyroid hormone tests were performed on every participant of the study at baseline. Levels of thyroid-stimulating hormone (TSH) and free thyroxine (FT^4^) were measured using a third-generation immunoenzymatic assay (Siemens, Deerfield, IL, USA) in serum obtained from centrifuged venous blood samples taken after an overnight fast. FT4 levels were only evaluated in participants with abnormal TSH levels. In this study, the reference range was 0.4-4.0 mIU/L for TSH and 0.8-1.9 ng/dL for FT4, which is similar to the reference range used in the National Health and Nutrition Examination Survey (NHANES III) (5) and recommended by Surks and cols. (2).

Levels of TPOAb were measured by electrochemiluminescence (Roche Diagnostics, Mannheim, Germany) and were considered positive when ≥ 34 IU/mL.

Participants in the ELSA-Brasil were classified into five categories of thyroid function according to levels of TSH and FT4 (when TSH was abnormal) and information regarding the use of medication to treat thyroid disorders: overt hyperthyroidism (low serum TSH and high FT4 levels or use of medication to treat hyperthyroidism); subclinical hyperthyroidism (low serum TSH, normal FT4 levels, and no use of thyroid medication or thyroid hormone); euthyroidism (normal TSH, normal FT4 levels and no use of thyroid medication or thyroid hormone); subclinical hypothyroidism (high TSH levels, normal FT4 levels, and no use of thyroid medication or thyroid hormone); and overt hypothyroidism (high TSH and low FT4 levels or use of levothyroxine to treat hypothyroidism). Therefore, subclinical thyroid disease was defined only in participants not taking medications to treat thyroid disorders.

Participants were excluded from the cohort when using medications that could affect the thyroid function such as amiodarone, carbamazepine, carbidopa, phenytoin, furosemide, haloperidol, heparin, interferon, levodopa, lithium, metoclopramide, propranolol, primidone, rifampicin, and valproic acid (28).

### Other variables

Age is presented as continuous or categorical (35-44, 45-54, 55-64, and 65-74 years) variables. Race was self-reported and categorized as white, brown, black, Asian, and indigenous. Anthropometric measures were obtained with standard techniques with the participants wearing light clothes. Body mass index (BMI) was calculated as weight divided by height squared (kg/m^2^). Hypertension was defined as the use of antihypertensive drug, systolic blood pressure ≥ 140 mmHg or diastolic blood pressure ≥ 90 mmHg. The diagnosis of diabetes was based on self-report by the participant or use of oral antidiabetic agents or insulin therapy, fasting plasma glucose ≥ 126 mg/dL, 2-hour post-prandial 75g glucose test ≥ 200 mg/dL, or glycosylated hemoglobin ≥ 6.5% (29). The diagnosis of dyslipidemia was considered in participants using cholesterol-lowering drugs or with an LDL-cholesterol level > 130 mg/dL.

### Statistical analysis

Data are expressed as mean and standard deviation (SD) for continuous variables and as numbers of individuals (frequency) for categorical variables. Subjects with positive and negative TPOAb were compared with the chi-square test. Stata 13.0 (Stata Corp., College Station, TX, USA) was used for all analyses (30). A p value < 0.05 was considered signiﬁcant.

## RESULTS

We analyzed 13,503 participants with measured serum TPOAb levels. Positive TPOAb was observed in 12% of the cohort (Table 1). In this group, 69% were women and the mean age was 52.6 ± 8.7 years.

**Table 1:** Characteristics of the cohort according to the presence or absence of antithyroperoxidase antibodies

	Antithyroperoxidase antibodies		p

	< 34 IU/mL n = 11,875	≥ 34 IU/mL n = 1,628	
Women (%)	6,206 (52.3)	1,124 (69)	< 0.0001
Age (SD) – years	51.8 (9.1)	52.6 (8.7)	0.001
Age strata (%)			0.003
35-44	2,742 (23.1)	312 (19.2)	
45-54	4,675 (39.4)	654 (40.2)	
55-64	3,265 (27.5)	490 (30.1)	
65-74	1,193 (10)	172 (10.6)	
Race (%)			< 0.0001
White	6,097 (52)	949 (58.8)	
Brown	3,316 (28.3)	403 (25)	
Black	1,885 (16.1)	217 (13.4)	
Asian	311 (2.7)	31 (1.9)	
Indigenous	126 (1.1)	14 (0.9)	
BMI (SD) – kg/m^2^	26.9 (4.7)	27.2 (4.9)	0.02
Waist circumference (SD) - cm	91.1 (12.7)	90.4 (12.7)	0.04
Hypertension (%)	4,112 (34.6)	525 (32.2)	0.057
Diabetes (%)	2,275 (19.2)	284 (17.4)	0.10
Dyslipidemia (%)	6,874 (57.9)	981 (60.3)	0.07
Smoking (%)			0.23
Never	6,743 (56.8)	928 (57)	
Past	3,553 (29.9)	507 (31.1)	
Current	1,578 (13.3)	193 (11.9)	
Alcohol use (%)			0.36
Never	1,240 (10.5)	185 (11.4)	
Past	2,326 (19.6)	331 (20.3)	
Current	8,294 (69.9)	1,112 (68.3)	
Thyroid function			< 0.0001
Subclinical hyperthyroidism (%)	154 (1.3)	18 (1.1)	
Overt hyperthyroidism (%)	73 (0.6)	17 (1)	
Subclinical hypothyroidism (%)	535 (4.5)	197 (12.1)	
Overt hypothyroidism (%)	537 (4.5)	451 (27.8)	
Euthyroidism (%)	10,560 (89)	941 (57.9)	

SD: standard deviation; BMI: body mass index.

BMI was slightly higher in the group with positive TPOAb (p = 0.02), whereas waist circumference was slightly higher in the group with negative TPOAb (p = 0.04). Other comorbidities (hypertension, diabetes, dyslipidemia, and smoking) were not associated with positive TPOAb.

**Figure 1:**
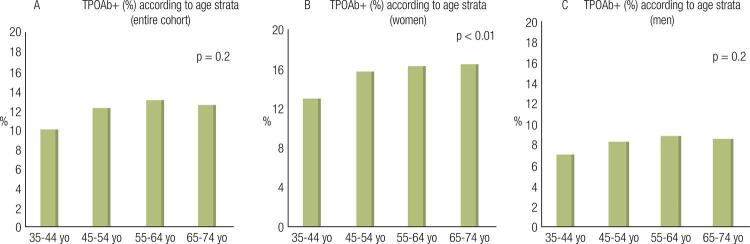
Distribution of percentages of antithyroperoxidase antibodies (TPOAb) according to age strata (A: Entire cohort, B: Women, C: Men). TPOAb+ – positive TPOAb; yo – years of age.

When stratified by age, the prevalence of positive TPOAb increased from the age of 35 to 64 years, although the prevalence was slightly lower in the elderly (p = 0.02). The same trend was noted in men (p = 0.02), while in women the prevalence increased linearly with age, from the age of 35 to 74 years (p < 0.001) (Figure 1).

**Figure 2:**
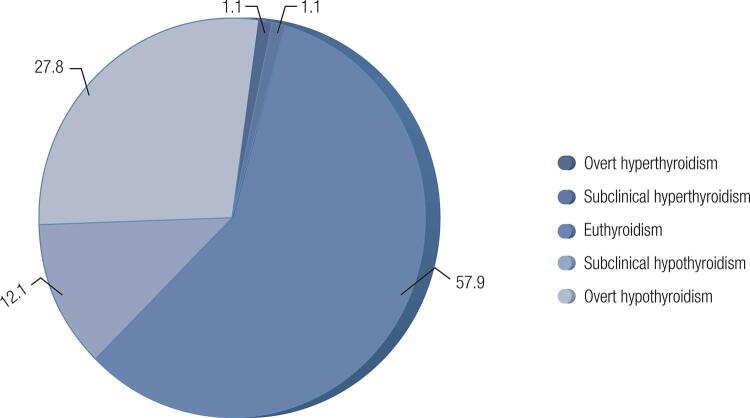
Distribution (%) of thyroid function in individuals with positive antithyroperoxidase antibodies.

When analyzed across the spectrum of thyroid function, 8.2% of the euthyroid population had positive TPOAb. Both overt and subclinical hypothyroidism represented the most common conditions associated with positive TPOAb, with a prevalence of 45.6% in participants with overt hypothyroidism and 27% in those with subclinical hypothyroidism (Figure 2). TPOAb was also present in 20% of the individuals with hyperthyroidism, including both subclinical and overt disease (Figure 2).

**Figure 3:**
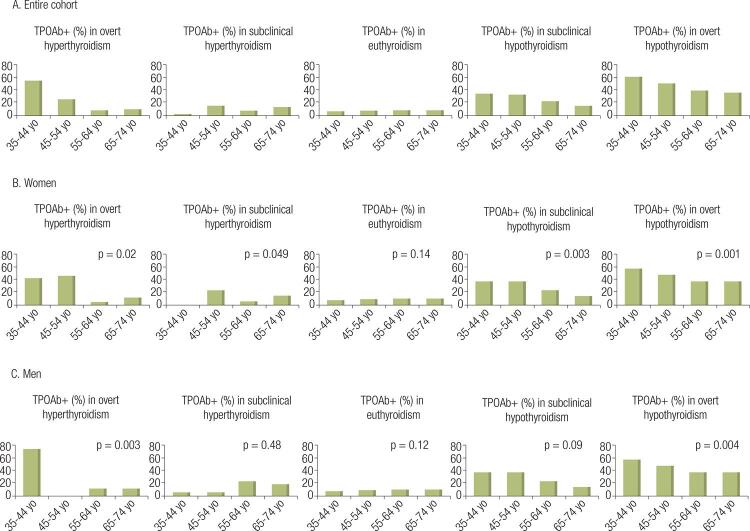
Distribution of the percentage of antithyroperoxidase antibodies (TPOAb) according to thyroid function, age strata, and gender (A: Entire cohort, B: Women, C: Men). TPOAb+ – positive TPOAb; yo – years of age.

In women, the entire spectrum of thyroid diseases was associated with the presence of TPOAb, while in men, only overt hyperthyroidism and overt hypothyroidism were associated with the presence of the antibodies (Figure 3).

When divided by Brazilian regions, there were significant differences in the prevalence of TPOAb among the cities. The distribution of TPOAb positivity among the cities was 37.7% in São Paulo, 12.7% in Porto Alegre, 11.3% in Rio de Janeiro, 21.9% in Belo Horizonte, 6.1% in Vitória, and 10.1% in Salvador.

## DISCUSSION

In our study of a large Brazilian population, positive TPOAb had a prevalence of 12% and was more common in women. This is the first cross-sectional study evaluating the prevalence of TPOAb in a large Brazilian adult cohort.

The largest data on this matter was collected by the National Health and Nutrition Examination Survey (NHANES III) between 1999 and 2002 and published in 2007 (5). In the survey, which represented the North American population, TPOAb was present in 13.0 ± 0.4% of the participants. In the NHANES III cohort (5), increased serum TPOAb concentrations were more prevalent in women than men and less prevalent in blacks than other ethnic groups, as seen in our study. However, in the North American cohort, the TPOAb concentration increased with age, which contrasts with our findings, since in our study the prevalence increased only in women and not in men.

Most studies have shown that the presence of TPOAb increases with age (5,25), which was not confirmed in our study. Aligned with our findings, Bjoro and cols. (24) have also observed that older people may not have higher TPOAb levels despite the presence of hypothyroidism. This could be due to atrophic degeneration of the thyroid gland caused by the autoimmune disease.

Another important prospective study from an Iranian group (25) has analyzed the prevalence and incidence of TPOAb. At baseline, the prevalence of TPOAb in that study was 12.8%, and the total incidence of TPOAb positivity was 7.1 per 1000 person-years of follow-up. That group evaluated the association between TPOAb positivity and the incidence of hypothyroidism over time. In our study, considering the spectrum of disease, we could see that the more hypothyroid the participants, the higher the prevalence of positive TPOAb (27% in subclinical hypothyroidism and 45.6% in overt hypothyroidism). Although our cross-sectional study did not determine the risk of TSH increase with positive TPOAb, such risk might be expected, as reported in other longitudinal studies (25,31-33), and will be estimated in the ELSA-Brasil cohort in the near future, when prospective data about the thyroid function of the participants are available.

One important topic when discussing autoimmune thyroid disease is the iodine status of the population. Brazil is considered an iodine-sufficient area since salt iodination is compulsory in the country. However, no studies have determined the exact urinary iodine concentration in the general Brazilian population (34). Some studies have shown that pregnant women may be at risk for iodine deficiency (35), while others have shown iodine excess among preschool children (36-38). Considering that the population analyzed in the ELSA-Brasil comprises adults with access to iodinated salt, we expected all the participants to be iodine-sufficient and the results to be compatible with those from iodine-sufficient countries.

ELSA-Brasil is not a population-based study. The study participants had a higher education level and income status than the overall Brazilian population. Although we recognize that the sampling strategy of the ELSA-Brasil limits the investigation in the domains of extremely poor and unemployed individuals, the racial, social, and regional diversity captured in the cohort allow an in-depth investigation of important health-related factors in Brazil. The applicability of estimates from the ELSA-Brasil study to the general Brazilian adult population is supported by their comparable prevalences of behavioral risk factors and chronic conditions. In the ELSA-Brasil, these variables were assessed with procedures similar to those used in the VIGITEL, an annual, telephone-based survey assessing behavioral risk factors of a representative sample of adults living in 27 state capitals and the Federal District in Brazil (39). Furthermore, the ELSA-Brasil included a large sample of participants from six different cities in Brazil. All measurements were obtained using the same protocol at all centers, with centralized training and under strict quality control. The difference in TPOAb prevalence among the cities is due to many variables. São Paulo accounts for the highest prevalence of positive TPOAb, but is the city with the highest rate of migration from other regions. Therefore, we cannot conclude that the different prevalences are affected by the region alone. Belo Horizonte, a region with an increased prevalence of goiter, also had a high prevalence of antibodies. The coastal cities had a lower prevalence of positive TPOAb, which could suggest an important role of iodine. Since the ELSA-Brasil was an epidemiologic cohort, we opted to evaluate the frequencies and prevalences in the country as a whole, instead of individualizing the analysis in regions, in order to prevent comparisons.

In conclusion, this descriptive report on the prevalence of positive TPOAb in Brazil showed that 12% of the population is TPOAb positive and that most of the individuals with positive TPOAb were white women. This finding is aligned with the prevalence of TPOAb positivity reported globally in iodine-sufficient areas.
